# Effect of phase response curve skew on synchronization with and without conduction delays

**DOI:** 10.3389/fncir.2013.00194

**Published:** 2013-12-11

**Authors:** Carmen C. Canavier, Shuoguo Wang, Lakshmi Chandrasekaran

**Affiliations:** ^1^Department of Cell Biology and Anatomy, Louisiana State University School of Medicine, Louisiana State University Health Sciences CenterNew Orleans, LA, USA; ^2^Neuroscience Center, Louisiana State University Health Sciences CenterNew Orleans, LA, USA

**Keywords:** synchrony, synchronization, pulsatile coupling, phase locking, phase resetting

## Abstract

A central problem in cortical processing including sensory binding and attentional gating is how neurons can synchronize their responses with zero or near-zero time lag. For a spontaneously firing neuron, an input from another neuron can delay or advance the next spike by different amounts depending upon the timing of the input relative to the previous spike. This information constitutes the phase response curve (PRC). We present a simple graphical method for determining the effect of PRC shape on synchronization tendencies and illustrate it using type 1 PRCs, which consist entirely of advances (delays) in response to excitation (inhibition). We obtained the following generic solutions for type 1 PRCs, which include the pulse-coupled leaky integrate and fire model. For pairs with mutual excitation, exact synchrony can be stable for strong coupling because of the stabilizing effect of the causal limit region of the PRC in which an input triggers a spike immediately upon arrival. However, synchrony is unstable for short delays, because delayed inputs arrive during a refractory period and cannot trigger an immediate spike. Right skew destabilizes antiphase and enables modes with time lags that grow as the conduction delay is increased. Therefore, right skew favors near synchrony at short conduction delays and a gradual transition between synchrony and antiphase for pairs coupled by mutual excitation. For pairs with mutual inhibition, zero time lag synchrony is stable for conduction delays ranging from zero to a substantial fraction of the period for pairs. However, for right skew there is a preferred antiphase mode at short delays. In contrast to mutual excitation, left skew destabilizes antiphase for mutual inhibition so that synchrony dominates at short delays as well. These pairwise synchronization tendencies constrain the synchronization properties of neurons embedded in larger networks.

## INTRODUCTION

A role has been proposed for synchronous oscillations in binding of sensory experiences (Singer, 1993) and attention (Fries et al., 2001). Synchronization that occurs between distal brain regions is almost always associated with oscillatory activity (Konig et al., 1995). This synchrony is achieved rapidly (Singer, 1999) and persists only transiently. The role of reciprocal coupling in synchronizing neural oscillators is supported by the observation that strong inter-hemispheric phase locking in the gamma frequency band with zero phase lag occurred in cat visual cortex could be disrupted by severing the corpus callosum (Engel et al., 1991). The inter-hemispheric conduction delays were on the order of 4–6 ms, which is about a sixth to a third of a gamma cycle. A role for altered synchronization tendencies in disease states (Uhlhaas and Singer, 2006) is supported by the observations that long distance synchronization is reduced in schizophrenia and epilepsy, whereas local synchronization in epilepsy is enhanced. Phase resetting theory (Glass and Mackey, 1988; Winfree, 1990; Ermentrout and Terman, 2010) is often used to study the synchronization tendencies of regularly spiking neurons. A phase response curve (PRC) shows how much an input advances or delays the next spike as a function of where in the cycle the input is applied. Type 1 PRCs (Hansel et al., 1995) are comprised of either all advances (for excitation) or all delays (for inhibition), whereas type 2 PRCs exhibit both advances and delays.

Neurons with type 1 PRCs tend not to synchronize via weak mutual excitation (Hansel et al., 1995; Ermentrout, 1996). Nonetheless, the ability of pulse-coupled leaky integrate and fire (LIF) and other oscillators with type 1 PRCs to synchronize due to strong mutual excitation is well known (Peskin, 1975; Mirollo and Strogatz, 1990). The PRC of this model at late phases has a strongly stabilizing slope due to the ability of an input to trigger a spike immediately on arrival at very late phases, which creates a linear “causal limit” region in the PRC. This region accounts for synchrony at zero delay (Canavier and Achuthan, 2010), and as we show in this study, also accounts for the existence of a gradual transition between synchrony and antiphase as the conduction delay is increased, regardless of PRC skew. In contrast, a critical role for PRC skew in networks of type 1 neurons connected by mutual synaptic excitation was demonstrated by Ermentrout et al. (2001). If the maximum resetting (of either sign) occurs in the first half of the cycle, the PRC is left skewed; on the other hand if it occurs in the right half, it is right skewed. If the right skew is increased, the tendency to approximately synchronize with small time lags is increased for pairs of type 1 neurons coupled via mutual synaptic excitation or electrical coupling (Pfeuty et al., 2003; Zahid and Skinner, 2009), and skewing the PRC toward the left stabilizes the antiphase mode. In contrast, the antiphase mode is stabilized by skewing the PRC to the right (Ladenbauer et al., 2012) for pair with type 1 mutual inhibition. There are several ways in which altering the conductances (Ermentrout et al., 2001, 2012; Pfeuty et al., 2003; Gutkin et al., 2005; Stiefel et al., 2009) can change the shape of a type 1 PRC for a regularly spiking neuron. Therefore, one way to quickly reverse the synchronization tendencies of neurons is to modulate the intrinsic ion channels that alter the PRC shape (Ermentrout et al., 2012), which could provide a switch to turn synchrony on and off rapidly.

Here we examine the effect of changing the skew of a type 1 PRC on the ability of pairs of neurons characterized by these PRCs to synchronize in the presence of conduction delays. We quantify the tendency of a network to synchronize using a global method that requires the identification of the unstable solutions comprising the boundaries between the attractive basins of the stable solutions, and compares the size of the sets of initial conditions, or basins of attraction, that lead to synchrony versus any other competing stable modes. In some cases we also use a local measure that infers the rate of convergence to synchrony in the neighborhood of a stable solution using the slopes of the PRC at the phases at which inputs are received with a possibly non-zero delay after spikes in the presynaptic neuron(s). The solution structure for pairs of coupled neurons with type 1 PRCs in the presence of conduction delays is highly dependent upon the skew of the PRC. In particular, right skew enhances the ability of mutually excitatory pairs to preserve synchrony in the presence of small delays, but diminishes that of inhibitory pairs. Overall, inhibitory synchrony (Van Vreeswijk et al., 1994; Wang et al., 2012) is much more robust to conduction delays. These results have implications for synchronization in larger networks as well (see Implications of Generic Modes for Larger Networks).

## MATERIALS AND METHODS

### WANG–BUZSAKI MODEL

The Wang and Buzsaki (1996) conductance-based model neuron has the following parameters unless otherwise noted. The reversal potentials *E*_Na_, *E*_K_, and *E*_L_ were set to 55, -90, and -65 mV, respectively and the capacitance was set to 1 μF/cm^2^. The maximal sodium (*g*_Na_), potassium (*g*_K_), and leak (*g*_L_) conductances were set to 35, 9, and 0.1 mS/cm^2^, respectively. *I*_stim_ is the applied current and was set at 1.0 μA/cm^2^. The synaptic current is given by *I*_syn_ = *g*_syn_*s*(*V* - *E*_syn_), where *g*_syn_ is the maximum synaptic conductance and *E*_syn_ is equal to -75 mV for inhibitory synaptic connectivity and equal to 0 mV for excitatory synaptic connectivity. The rate of change of the gating variable s in units of ms^-^^1^ is d*s*/d*t* = 6.25(1 - *s*)/[1 + exp(-*V*_pre_/2)] - *s*/τ_syn_, where *V*_pre_ is the voltage of the presynaptic cell, and τ_syn_ is the synaptic decay time constant of 1.0 ms.

### MEASUREMENT OF PRC IN ISOLATED WANG–BUZSAKI NEURONS

**FIGURE 1:**
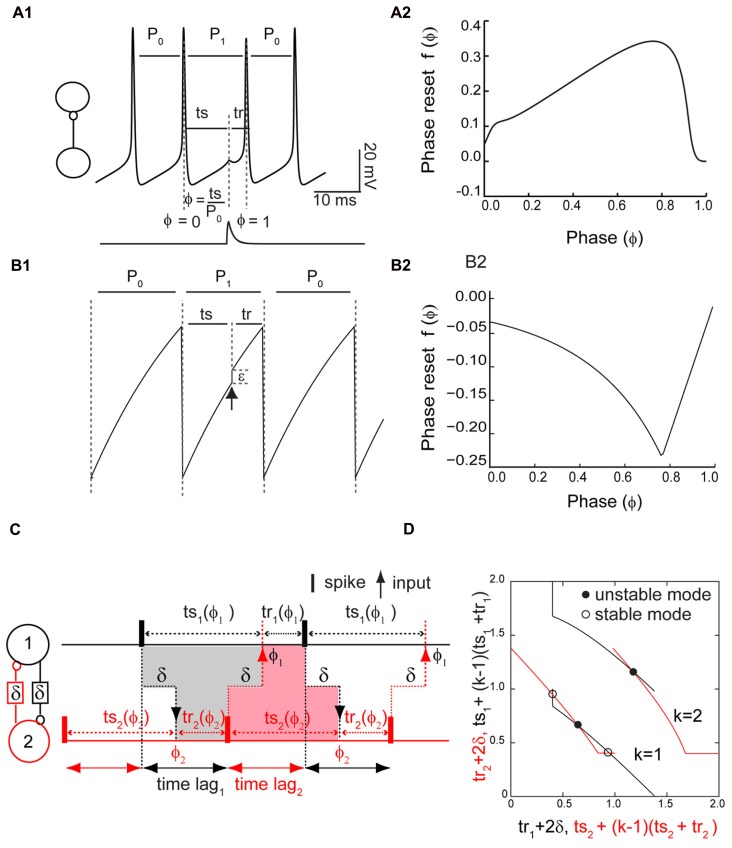
**FIGURE 1.** **Phase response curve (PRC) measured in isolated neurons and used to predict network activity.** **(A1)** PRC measurement in a Wang–Buzsaki model neuron. Inset shows open loop configuration without feedback. A perturbation in the form of a synaptic conductance waveform evoked by a single spike in the presynaptic neuron (lower trace) is applied at a phase of ϕ = *t*s/*P*_0_, and the phase resetting *f*(ϕ) is the normalized change in cycle length *f*(ϕ) = (*P*_1_ - *P*_0_)/*P*_0_. Alternatively, the perturbed cycle length *P*_1_ is equal to the sum of the stimulus (*t*s) and recovery (*t*r) intervals. **(A2)** Typical PRC for an inhibitory input to a Wang–Buzsaki neuron with *g*_syn_ = 0.35 mS/cm^2^, τ_syn_ = 1 ms, *I*_stim_ = 2.0 μA/cm^2^, and otherwise as the Methods. **(B1)** PRC measurement in a leaky integrate and fire model neuron. An instantaneous increment in membrane potential (black arrow) either advances the phase or immediately causes the neuron to reach threshold. **(B2)** Typical PRC for leaky integrate and fire neuron. Beyond a phase of about 0.8, a spike is triggered immediately by the input. In this “causal limit” region, the resetting is equal to ϕ - 1. **(C)** Stimulus and recovery intervals in the network. Inset shows closed loop configuration with feedback. In an alternating firing pattern, each spike affects the timing of the very next spike (*k* = 1) in the same neuron via a feedback loop through the partner neuron. In a phase-locked mode with constant firing intervals, the gray shaded area indicates that the stimulus interval in neuron 1 is equal to the recovery interval in neuron 2 plus twice the delay δ, and the pink shaded area illustrates a similar constraint for the stimulus interval in neuron 2. The time lags, or firing intervals between neurons, can be inferred from the stimulus and recovery intervals. **(D)**. Predicting closed loop modes with open loop data. Plotting the algebraic combination of intervals with quantities that must be equal in a phase-locked mode on the same axis ensures that the intersections represent the stimulus and recovery intervals in phase-locked modes. The delay was 20% of the intrinsic period *P*_0_. The axes are all normalized by the intrinsic period of the component oscillators and therefore dimensionless.

**Figure 1A1** shows the measurement of the PRC for a Wang–Buzsaki model neuron where the input is the synaptic conductance waveform (**Figure 1A1**, bottom trace) that results from a spike in the presynaptic neuron. The phase ϕ is 0 at an upward crossing of a predetermined threshold (here -14 mV), and the phase ϕ at which a stimulus is received is *t*s/*P*_0_, where *P*_0_ is the intrinsic period and *t*s = ϕ*P*_0_ is the stimulus interval, defined as the interval between the time of the action potential and the receipt of an input. The recovery interval *t*r is defined as the interval between the receipt of an input by a neuron and the next action potential in the same neuron: *t*r = *P*_0_ - *t*s + *P*_0_*f*(ϕ), where the phase resetting *f*(ϕ) is given by the normalized change in the cycle length that contains the perturbation *f*(ϕ) = (*P*_1_ - *P*_0_)/*P*_0_. In this study we do not focus on second order resetting that is evidenced by changes in length of the second cycle following the perturbation, but in some cases the second order phase resetting must be considered (Oprisan et al., 2004; Maran and Canavier, 2008; Woodman and Canavier, 2011). A positive resetting signifies a phase delay and a negative resetting signifies a phase advance. **Figure 1A2** shows a typical PRC for the Wang–Buzsaki model, consisting of all delays in response to an inhibitory synaptic input.

### LEAKY INTEGRATE AND FIRE NEURON MODEL PRC

The LIF model is given by d*V*/d*t* = -γ*V*(*t*) + *S*_0_, where *V*(*t*) is the membrane potential, γ is the magnitude of the leak, and *S*_0_ is the applied current. When *V*(*t*) = 1 the neuron is presumed to fire and *V*(*t*) is reset to 0 (**Figure 1B1**). Following the methods of Peskin (1975) and Mirollo and Strogatz (1990), the neurons are instantaneously pulse-coupled such that an input depolarizes the membrane by a fixed amount ε or brings the membrane potential to threshold, whichever among the two values is less. For two coupled neurons i and j, when *V*_i_(*t*) = 1, meaning one neuron reaches spike threshold, then the potential in the partner is set to *V*_j_(*t*) = min [1,*V*_j_(*t*)+ε], j ≠ i, meaning that inputs that occur late within the cycle can immediately trigger a spike. At an initial condition of *V*(0) = 0, we can explicitly solve for the voltage such that *V*(*t*) = (*S*_0_/γ)(1 - e^-^^γ^*^t^*). From this expression, solving for the elapsed time (*t*s) to reach a given value of voltage *V*(*t*), we obtain *t*s = (1/γ) ln{*S*_0_/[*S*_0_ - γ*V*(*t*)]}. The intrinsic period of the oscillator is the elapsed time required to reach *V*(*t*) = 1, which is *C*/γ, where *C* = ln[*S*_0_/(*S*_0_ - γ)]. We can solve for the phase advance due an instantaneous jump from *V*_j_(*t*) to *V*_j_(*t*) + ε by taking the difference between the elapsed time required to reach *V*_j_(*t*) corresponding to a given phase ϕ = *t*s γ/*C* and the elapsed time required to reach *V*_j_(*t*) + ε in the absence of a perturbation. For *V*_j_(*t*) + ε < 1, this difference is equal to (-1/γ){ln[(*S*_0_ - γ)/(*S*_0_ - γεe^C^^ϕ^)] - 1}, which is then normalized by the intrinsic period in Eq. 1. For *V*_j_(*t*) + ε ≥ 1, the resetting is limited by the fact that an input cannot advance the next spike time to a time before the neuron receives an input, so the phase is advanced by exactly the normalized time remaining until the next input (1 - ϕ), with a sign reversal due to our definition of phase resetting in which advances are negative. The resetting at 0 and 1 are not the same because the effect of an input is assumed to end when a spike is produced; a more physiological model for coupling would assume any excess charge beyond that required to evoke a spike is applied in the next cycle, causing an advance in that cycle, which at a phase of 1 would equal the resetting at a phase of 0.

Given the constraints on how much a spike can be advanced, the phase resetting (see **Figure 1B2**) is given by

(1) $$f\left(\Phi \right)=-\min\left(\left|\left(-1/C\right)\,\left\{I\,n\,\left[\left(S_{0}-\gamma \right)/\left(S_{0}-\gamma \,\varepsilon \,e^{C\,\Phi }\right)\right]-1\right\}\right|,\left|\Phi -1\right|\right)$$

The negative sign in Eq. 1 was necessary to make the sign of the PRC consistent with the convention used in this work. The PRC in **Figure 1B2** was calculated directly from Eq. 1. This non-physiological feature by which an input instantaneously triggers a spike introduces a linear region in the PRC at late phases in which the phase advance is ϕ - 1, exactly equal in magnitude and opposite in sign to the fraction of the cycle remaining when the input is applied. Since this limit is imposed by causality, we call this linear region the causal limit region of the PRC.

### PREDICTION OF NETWORK ACTIVITY USING PHASE RESETTING

The assumptions required to apply the stimulus and recovery intervals measured in isolated neurons with no feedback to the closed loop circuit are simply that the spikes remain essentially the same in the presence of feedback, and that the effect of each perturbation dies out within a single network period after it is received. Detailed stability calculations are given in Woodman and Canavier (2011). We use a method (Woodman and Canavier, 2011; Wang et al., 2012) very similar to the spike time difference method (Acker et al., 2003) with the advantage that it is easily extendable to longer conduction delays.

Our method takes advantage of the algebraic relationship shown in **Figure 1C** between the stimulus and recovery intervals in one to one phase-locked periodic modes. The stimulus interval (in the absence of any second order resetting) is simply *P*_0_ϕ as described above, and the dependence of the recovery interval on the phase was determined using the phase resetting protocol also described above. The key idea (Woodman and Canavier, 2011) is that there is a feedback loop through which a spike in one neuron influences, after a conduction delay, the timing of a spike in its partner, and this spike in turn, after another conduction delay, affects the timing of a spike in the original neuron. The duration of this feedback loop is always the sum of the two delays plus the recovery interval in the partner. For short equal conduction delays, the duration of this feedback loop is exactly the stimulus interval in the original neuron, as illustrated in **Figure 1C**. This condition must be met with respect to both the stimulus interval in neuron 1 (**Figure 1C**, gray shaded area) and the stimulus interval in neuron 2 (**Figure 1C**, red shaded area), so there are two symmetric criteria that must both be satisfied in order to establish a periodic one to one phase locking. However, longer feedback loops are also possible, in which the duration of the feedback loop is still equal to twice the conduction delay plus the recovery interval in the partner, but one or more spikes occur in the original neuron before the feedback from a given spike is received. The duration of the feedback loop in the original neuron is then equal to the stimulus interval in the original neuron plus *k* - 1 network periods *P*_N_, where the parameter *k* - 1 is the number of spikes that occur before the feedback loop is closed, and the network period is the sum of the stimulus and recovery intervals associated with any given input phase.

The stimulus and recovery intervals measured using the PRC protocol can be plotted for each isolated neuron with the axes arranged as in **Figure 1D** so that the intersection points meet both criteria for the duration of the feedback loop described above that must be satisfied in a periodic one to one locking by the stimulus and recovery intervals in each neuron. The observable time lags between neural firings can be calculated using the algebraic relationships shown in **Figure 1C** (Woodman and Canavier, 2011). In addition to the phasic relationships within a periodic mode, we also need to know the stability of each mode. The stability can also be read from the graph in **Figure 1D** (Wang et al., 2012), at least for *k* = 1. The stability criterion for the *k* = 1 mode mandates that if the absolute value of the slope of the black curve is greater than the slope of the red curve at an intersection, then that intersection is stable, hence a steeper black curve at the intersection point guarantees stability. The derivation follows from the stability criterion for modes with *k* = 1, which is -1 < [1 - *f*′(ϕ_1_)][1 - *f*′(ϕ_2_)] < 1 where *f*′(ϕ_1_) and *f*′(ϕ_2_) are slopes of the PRC evaluated at the phase locking points of ϕ_1_ and ϕ_2_. Stability is guaranteed if the slope of the PRC at both locking points is positive and <2. Since ts depends only on phase, and *t*r depends on both the phase and the phase resetting, algebraic manipulation reveals that the slope of the black curve for neuron 1 for *k* = 1 is [*f*′(ϕ_1_) - 1]^-^^1^ and the slope of the red curve for neuron 2 for *k* = 1 is [*f*′(ϕ_2_) - 1]. Dividing all terms in the stability criterion by [1 - *f*′(ϕ_1_)] and considering the cases for which [1 - *f*′(ϕ_1_)] is positive or negative gives the stability criterion in terms of the relative steepness of the slopes. For *k* = 2, the stability criterion is -1 < [1 - *f*′(ϕ_1_) - *f*′(ϕ_2_)] < 1. For higher values of *k*, the appropriate stability criterion must be applied (Woodman and Canavier, 2011).

## RESULTS

### TWO LIF NEURONS PULSE COUPLED BY EXCITATION TRANSITION GRADUALLY BETWEEN SYNCHRONY AND ANTIPHASE AS THE CONDUCTION DELAY IS INCREASED

**FIGURE 2:**
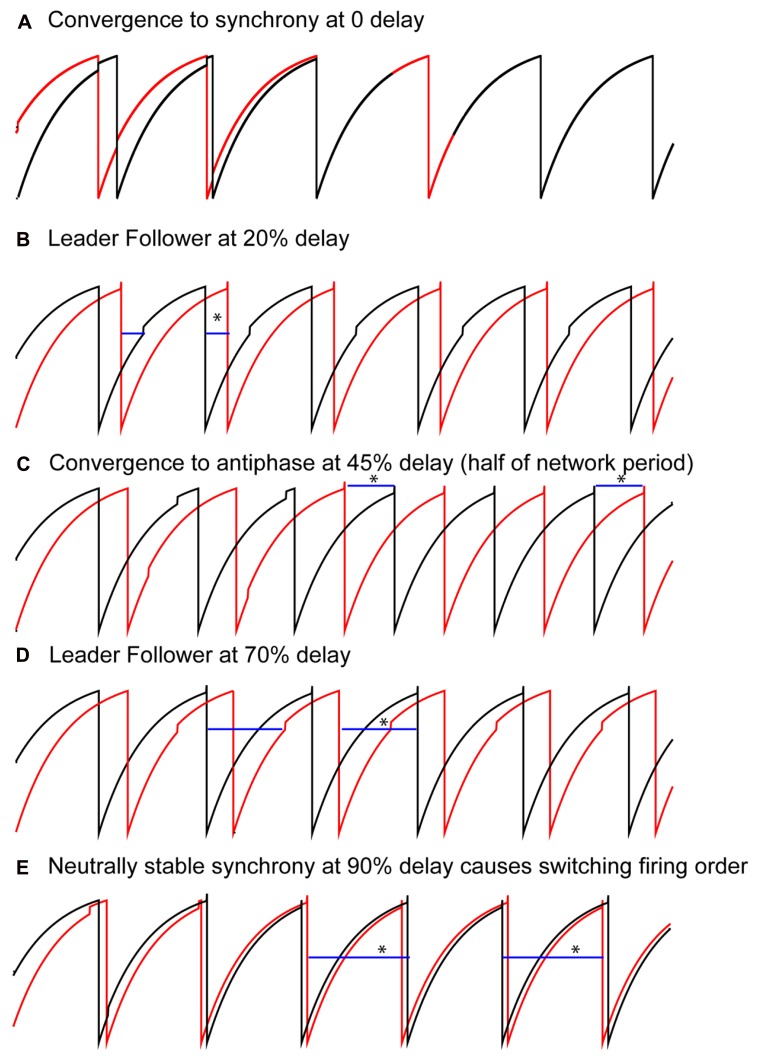
**FIGURE 2.** **Typical patterns observed in a pair of pulse-coupled LIF oscillators as the delay is varied.** Blue horizontal bars show the conduction delay. **(A)** For short delays, all initial conditions converge to synchrony. **(B)** For short delays, the shorter time lag (asterisk over blue bar indicating delay) between one neuron (black curve) and the other (red curve) is exactly equal to the delay. Note that the other time lag is not equal to the delay. **(C)** For midrange delays antiphase is observed in which each time lag (asterisks) is equal to the delay. This is not generic for antiphase, but rather a special case as explained in the text. **(D)** For longer delays, again only one time lag is equal to the delay, this time the longer time lag (see asterisk). **(E)** For delays almost equal to an intrinsic period, the trajectories do not converge to synchrony, instead the neurons switch their firing order on each cycle. The delays are equal to the time lags (asterisks). Parameters for the coupled LIF are γ = 0.9, *S*_0_ = 1, ε = 0.05.

Solutions that were obtained as the conduction delay was varied in pairs of LIF model neurons coupled via excitatory pulses are shown in **Figure 2**. With no delay, all initial conditions converged to synchrony (**Figure 2A**), as expected (Peskin, 1975; Mirollo and Strogatz, 1990). For delays >0 but up to about 40% of the intrinsic period, a “leader–follower” mode was obtained in which the smaller time lag between the firing of the two neurons was equal to the delay (second blue bar in **Figure 2B**). This mode is observed because the follower fires exactly when the delayed input from its partner arrives, but the leader does not fire immediately upon receiving an input from the follower. Convergence occurs within a single cycle for reasons explained below. The lack of robustness of synchrony mediated by excitatory pulse coupling to delays was also expected (Ernst et al., 1995). For delays equal to about 40–50% of the intrinsic period (see **Figure 4C**), an exact antiphase mode was obtained (**Figure 2C**) in which the time lags are each equal to half the network period, and because each neuron fired immediately upon receiving an input, the delays (horizontal blue bars) were exactly equal to the time lags. For delays equal to about 50–85% of the network period, we again obtained a leader–follower mode in which one neuron, but not the other, fired immediately after the delayed input from its partner arrived (second blue bar in **Figure 2D**). In this case the longer of the two time lags is equal to the delay, but convergence generally does not occur within one cycle. Finally, for delays longer than 90%, a nearly synchronous mode emerged in which the firing order of the two neurons switched on every cycle (leapfrog mode in Maran and Canavier, 2008; Oh and Matveev, 2008).

**FIGURE 3:**
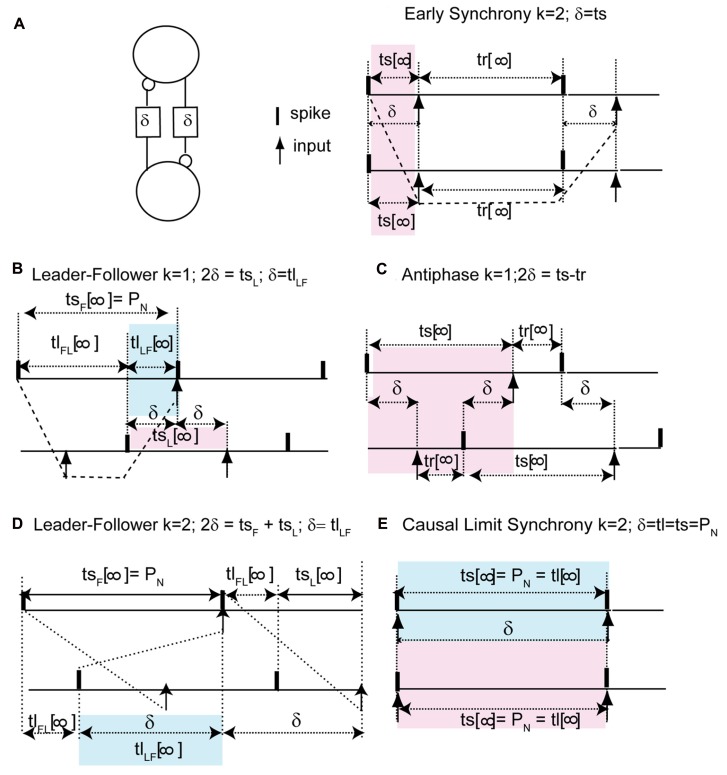
**FIGURE 3.** **Five generic modes in identical, identically pulse-coupled LIF oscillators with identical conduction delays.** Pink shaded regions show relationship of delay to the phase, blue show the relationship to the time lags. **(A)** For a synchronous mode and identical oscillators with short delays, the stimulus interval *t*s in each oscillator is equal to the delay δ. The dashed lines show that the first spike in the top neuron does not affect the timing of the very next spike in the same neuron, but rather the one after that, so *k* = 2. **(B)** In the leader–follower mode for *k* = 1, the stimulus interval in the follower (top) is equal to the network period, the time lag between the firing of the leader and that of the follower is equal to the delay, and the stimulus interval in the leader is twice the delay. The dashed lines show that the first spike in the top neuron does affect the timing of the very next spike in the same neuron, so *k* = 1. **(C)** Antiphase. For identical oscillators, both stimulus intervals are equal to twice the delay plus the recovery interval. **(D)** In the leader–follower mode for *k* = 2, the sum of the stimulus intervals is twice the delay, hence their average gives the delay. **(E)** Causal limit synchrony for long delays. In this case the stimulus intervals in both neurons are equal to the delay as in **(A)**, but because the recovery intervals are equal to 0, the stimulus intervals are also equal to both the network period and the delay. The delay is also equal to one time lag if the other is considered to be 0.

We can understand how the modes in **Figure 2** arise by examining how the delays alter the generic periodic solutions for two identical, identically coupled oscillators in which the receipt of an input at late phases can immediately trigger a spike. We consider as generic only 1:1 modes, in which no oscillator fires twice in a row before the other oscillator fires. The inset in **Figure 3** shows two oscillators coupled with equal conduction delays. **Figure 3** shows a schematic representation of the generic modes: the two oscillators can fire together in exact synchrony (**Figures 3A, E**), they can alternate in exact antiphase with the same time lags (**Figure 3C**), or they can fire alternately with different intervals between spikes (**Figures 3B, D**). The meaning of the integer *k* can be better understood by observing the paths marked by dashed lines in **Figures 3A, B**. The path begins with a spike in the top neuron and shows whether the timing of that spike affects the timing of the next spike in the spike neuron via the feedback loop through the other neuron. **Figure 3A** shows that after one delay, an input is received by the other neuron, then one recovery interval later the other neuron spikes, then after one more delay an input is received by the first neuron. This input arrives too late to affect the timing of the very next spike in the first neuron, but will affect the timing of the second, so *k* = 2. On the other hand, the dashed lines in **Figure 3B** show that the first spike in the top neuron does affect the timing of the very next spike in the same neuron via the feedback loop though the other neuron, so *k* = 1 for this case.

The pink shaded areas in **Figure 3** show the relationship of the stimulus intervals to the conduction delay, and the infinity symbol represents the steady value of the intervals in a periodic mode after all transients have decayed. This relationship is important because it allows us to predict the phase that at which inputs will be receive in a given model directly from the value of the conduction delay. For synchrony at both early (**Figure 3A**) and late phases (causal limit synchrony, **Figure 3E**), the conduction delay is equal to the stimulus interval in each neuron (δ = *t*s = ϕ*P*_0_). Therefore the phase at which an input is received in the synchronous modes is always equal to the normalized delay (ϕ = δ/*P*_0_). We refer to **Figure 3E** as causal limit synchrony because a spike is triggered immediately when the delayed input is received; this is not the case in **Figure 3A**. The pink shaded area in **Figure 3B** shows that the delay is half the stimulus interval (*t*s_L_) for the leader, so the phase at which an input is received is half the normalized delay. The pink shaded area in **Figure 3C** shows that in the antiphase mode the stimulus interval in one neuron is equal to twice the delay plus the recovery interval in the other neuron. There is no pink shaded area in **Figure 3D** because there is no integral relationship between one stimulus interval and the conduction delay; instead the delay is half the sum of the two stimulus intervals. This result is obtained by noting *t*s_F_ + *t*l_FL_ + *t*s_L_ = *t*l_FL_ + 2δ and canceling the time lag term tl_FL_.

The blue shaded areas in **Figure 3** show the relationship of the time lags observed in each mode to the conduction delay. **Figures 3B, D** show that for leader–follower modes with *k* = 1 and 2 respectively, the delay is equal to the time lag between the leader and the follower (*t*l_LF_), meaning that a spike is triggered in one neuron, but not the other, immediately when a delayed input is received. The blue shaded area in **Figure 3E** shows that in the causal synchrony mode, a spike is triggered in both neurons immediately upon receipt of the delayed input, and consequently both stimulus intervals as well as the network period are equal to the conduction delay. These relationships are a direct consequence of the ability of a delayed input to immediately trigger a spike upon its arrival.

**FIGURE 4:**
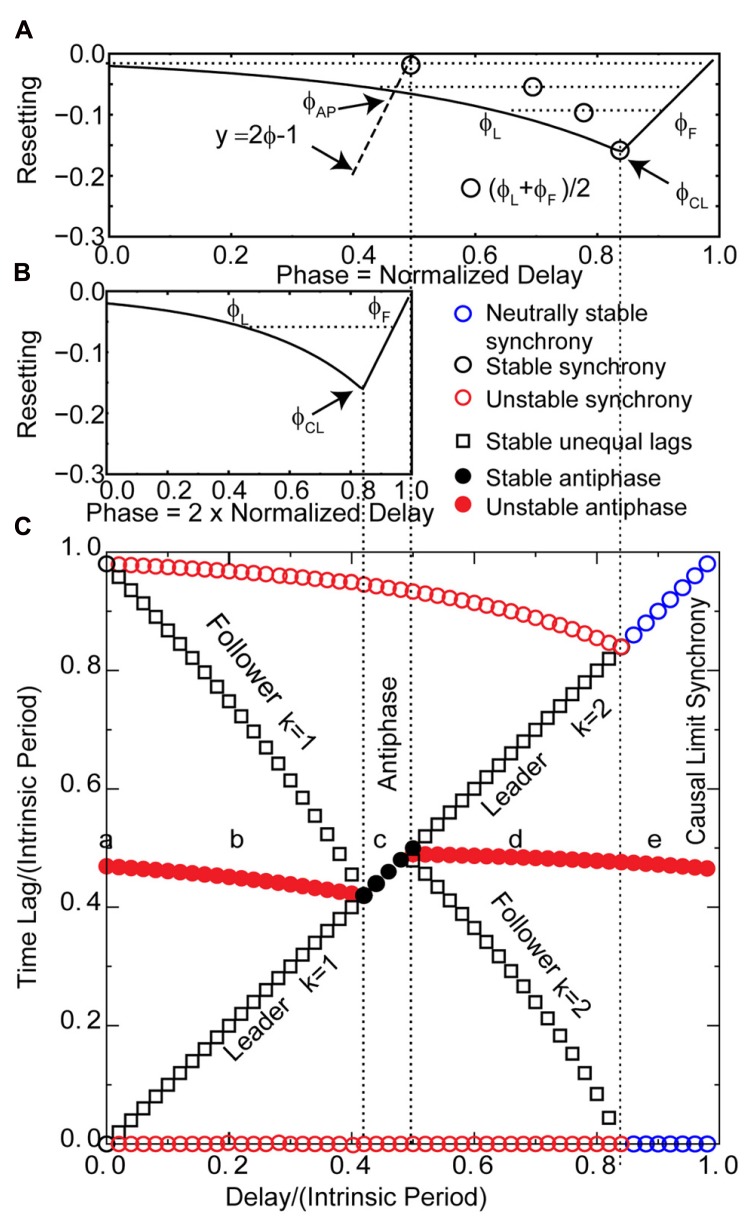
**FIGURE 4.** **Predicting the solution structure for pulse-coupled LIF pairs.** **(A)** Phase response curve for a leaky integrate and fire neuron with parameters as in **Figure 2**. The unstable branch is to the left of ϕ_CL_. The stable, causal limit branch of the PRC is to the right of ϕ_CL_, and neurons receiving an input on this branch fire immediately upon receipt of the input. The input phases in leader–follower mode ϕ_L_ and ϕ_F_ lie on the left and right branches, respectively, and must have equal phase resetting *f*(ϕ_L_) = *f*(ϕ_F_) as indicated by horizontal dotted line. Open circle denotes the average of ϕ_L_ and ϕ_F_. The vertical dotted lines from **(A)** to **(C)** give the boundaries in **(C)** of the leader–follower mode for *k* = 2 and the causal limit synchrony region. The dashed line labeled *y* = 2ϕ - 1 give the input phase for the antiphase mode ϕ_AP_ with zero delay. If the center of the PRC (open circles) falls to the right of this line, the leader–follower *k* = 1 branch exists. **(B)** The PRC is replotted at half scale to show the generic relationships between the normalized stimulus interval, phase (ϕ = *t*s/*P*_0_) and the normalized delay (δ/*P*_0_) in the leader–follower mode for *k* = 1 and the antiphase mode. The phase ϕ_L_ at which the leader receives an input for the *k* = 1 leader–follower mode is twice the normalized delay (ϕ_L_ = *t*s_L_/*P*_0_; *t*s_L_ = 2δ, see **Figure 3B**, so ϕ_L_ = 2δ/*P*_0_). The follower receives an input at phase ϕ_F_ and fires immediately. This leader–follower mode ceases to exist when twice the normalized delay value reaches ϕ_CL_; beyond that point, the antiphase mode gains stability. In this antiphase mode, both neurons receive an input at the same phase (see **Figure 3C**) on the right stable branch indicated in **(B)**, and fire immediately upon receiving the input. On this branch, the normalized stimulus interval for each neuron is equal to twice the normalized delay. **(C)** Predicted solution structure as delays are varied for two neurons coupled via the PRC in **(B1)**. The two time lags between the firings of the two neurons are represented by a pair of red symbols (unstable mode), a pair of black symbols (stable mode) or a pair of blue circles (neutrally stable mode). In an antiphase mode, both time lags are the same, which is indicated by a filled symbol. For synchrony, one time lag of each pair must be 0 and the other is equal to the network period. The leader–follower mode for *k* = 1 persists for normalized delays up to ϕ_CL_/2 indicated by the leftmost vertical dotted line from **(B)** to **(C)**. In the stable antiphase mode for normalized delays from ϕ_CL_/2 to 0.5, both time lags are exactly equal to the delay (see **Figure 3C**). The leader–follower mode re-establishes itself for normalized delays >0.5 and persists until the normalized delay reaches ϕ_CL_. This leader–follower mode has a *k* = 2 and the normalized delay is equal to (ϕ_L_ + ϕ_F_)/2 (see **Figure 3D**). Neutrally stable causal limit synchrony (see **Figure 3E**) is observed starting at normalized delays greater than ϕ_CL_ indicated by rightmost vertical dashed line from **(A)** to **(C)**. Note the diagonal line formed by the black and blue symbols indicates that in every stable or neutrally stable mode, at least one time lag is equal to the delay. The lower a, b, c, d, and e in **(C)** correspond to the labels of the same letter in **Figures 2 and 3**.

The overall picture given in **Figure 4** with respect to the generic modes is as follows. There are three solution branches, corresponding to synchrony, leader–follower and antiphase. Synchrony is stable (a) at zero delay (open black circles) but in region (b) splits into an unstable synchronous branch (pairs of open red circles) and a stable leader–follower branch (open black squares). In the same regions, antiphase is unstable (solid red circles). Unlike the weak coupling approach, the network period is not equal to the intrinsic period, because the network period includes a non-negligible contribution from the phase resetting in the circuit. Therefore, the normalized time lag is not 0.5 for antiphase because the normalization is by the intrinsic period and not the network period. At the start of the region labeled (c), the stable leader–follower branch and the unstable antiphase branch coalesce into a stable antiphase branch. Therefore, the stable leader–follower branch allows for a gradual transition between synchrony and antiphase as the delay is lengthened. At the start of the region (d) these two branches again diverge with antiphase losing stability and leader–follower regaining existence. At the start of region (e) the stable leader–follower and unstable synchronous branches merge into neutrally stable causal limit synchrony (open blue circles). Neutrally stable synchrony implies that near synchronous solutions, like the one shown in **Figure 2E**, do not converge to synchrony.

The diagonal line of symbols in **Figure 4C** indicates that for every predicted one to one stable or neutrally stable phase-locked mode, one or both time lags are equal to the delay. We will show that the PRCs in **Figures 4A, B**, along with the understanding of the generic modes presented in **Figure 3**, can explain the relationship of these time lags to the delay as well as why solution branches coalesce, diverge, or change stability. The key characteristic of the PRCs in **Figures 4A, B** is that they have two branches, a left branch with a negative destabilizing slope and a right branch with a maximally stabilizing slope to the right of the phase marked ϕ_CL_, which is the causal limit (CL) region described in Section “Materials and Methods.” Inputs received at phases in the causal limit region immediately trigger a spike.

### WHY IS EXACT SYNCHRONY STABILIZED BY THE CAUSAL LIMIT REGION OF THE PRC AND DISRUPTED BY CONDUCTION DELAYS?

At zero delay, indicated by the point labeled “a,” there is a stable synchronous solution (black circles in **Figure 4C**) and an unstable antiphase solution (solid red circle). For the synchronous solution, **Figure 3A** shows that both neurons receive an input at a phase equal to the normalized delay. Synchrony at zero delay is a special case because *k* = 1 for that case, and the relevant stability criterion for synchrony with no delay depends upon the slope of the PRC at the two ends, *f*′(0^+^) and *f*′(1^-^). Specifically for synchrony stability requires that -1 < [1 - *f*′(0^+^)][1 - *f*′(1^-^)] < 1 where the + and - superscripts indicate the limit from the right and left, respectively (Oprisan and Canavier, 2001; Achuthan and Canavier, 2009). The quantity [1 - *f*′(0^+^)][1 - *f*′(1^-^)] is a scaling factor that operates in the vicinity of synchrony and multiplies the phasic deviation from synchrony on one cycle to give the deviation on the next cycle.

If infinitesimally small delays are introduced, each spike no longer affects the timing of the very next spike in the same neuron via the feedback loop through the partner (**Figure 3A**). Instead, the effect is felt on the second spike after the spike that triggered the input, so *k* = 2 and the stability criterion becomes -1 < [1 - *f*′(0^+^) - *f*′(0^+^)] < 1 (Woodman and Canavier, 2011). For the negative slopes just to the right of zero, the scaling factor 1 - *f*′(0^+^) - *f*′(0^+^) is >1, resulting in deviations from synchrony that grow and render synchrony unstable. The major effect is not the change in the form of the stability criterion, but rather the loss of the stabilizing slope at a phase just to the left of one (1^-^), where the slope is nearly 1 so the scaling factor is nearly 0. The bottom line is that the slope of the left branch of PRC for excitation does not favor synchrony at short delays; therefore, zero time lag synchrony with mutual excitation is not robust to delays for this PRC shape. Since the stimulus interval is equal to the delay, the normalized delays and input phases on the PRC are numerically equal and synchrony remains unstable along the left branch of the PRC in **Figure 4A** until the normalized delay exceeds ϕ_CL_ (blue circles in region including the label e in **Figure 4C**). The neutrally stable causal limit branch emerges at that point with one time lag equal to the delay and the network period as shown in **Figure 3E**. Recall that the scaling factor that determines stability is 1 - *f*′(ϕ_1_) - *f*′(ϕ_2_) for *k* = 2. Both input phases are the same (ϕ_1_ = ϕ_2_) and fall on the causal limit line with a slope of 1 [*f*′(ϕ_1_) = *f*′(ϕ_2_) = 1]. Therefore, the scaling factor that determines whether perturbations from synchrony grow or decay is equal to -1. This implies that synchrony is neutrally stable, which means that perturbations do not decay; also the negative sign of the scaling factor guarantees that the firing order switches on every cycle preventing convergence to exact synchrony as shown in **Figure 2E**.

### WHEN DO YOU GET UNEQUAL TIME LAGS THAT TRANSITION BETWEEN SYNCHRONY AND ANTIPHASE?

In the leader–follower mode shown in **Figure 2B**, the follower neuron (red trace) but not the leader (black trace) fires immediately upon the delayed receipt of an input (see **Figure 3B**), thus its phase locking point lies in the causal limit region of the PRC. This particular mode has a conduction delay of 20% of the period, and is indicated by the open circles in the predictive plot for *k* = 1 in **Figure 1D** as well as by the black squares above and below the letter b in **Figure 4C**. As illustrated schematically in **Figure 3B**, one time lag (*t*l_LF_) is equal to the delay δ, and the stimulus interval for the leader (*t*s_L_) is exactly twice the delay. Therefore the PRC in **Figure 4B** is plotted so that the normalized stimulus intervals for the leader (the phase ϕ_L_) line up with the corresponding normalized delay (half the stimulus interval). For each ϕ_L_ on the left branch, the leader–follower mode for *k* = 1 exists if there is a corresponding ϕ_F_ point on the right, causal limit branch with the same resetting value, as illustrated by the horizontal dashed line. This leader–follower solution branch ends at ϕ_CL_ and coincides with the stabilization of the antiphase mode. The ability of a delayed input to immediately trigger a spike guarantees stable solutions for which the time lag is equal to twice the delay (see caption of **Figure 3D**) and enables near synchrony with short time lags. For *k* = 1, the scaling factor for deviations from the phase-locked mode is [1 - *f*′(ϕ_F_)][1 - *f*′(ϕ_L_)], which is 0 given that *f*′(ϕ_F_) is 1, so convergence is rapid.

The defining characteristic of the antiphase mode (**Figures 2C and 3C**) is that the two time lags are equal, the two stimulus intervals are equal and the two recovery intervals are equal. Each stimulus interval is also equal to the recovery interval plus twice the delay: *P*ϕ_AP_ = *P* - *P*ϕ_AP_ + *Pf*(ϕ_AP_) + 2δ. This implies that the phase at which an input is received in the antiphase mode is ϕ_AP_ = [1 + *f*(ϕ_AP_)]/2 + δ/*P*. For zero delay, the intersection of the line *y* = 2ϕ - 1 with the PRC occurs at ϕ_AP_, because on that line ϕ = [1 + *f*(ϕ)]/2. In **Figure 4**, for normalized delays less than ϕ_CL_, the phase corresponding to the antiphase mode falls on the left, unstable branch of the PRC. The stability of antiphase at zero delay is critical: the stability of this mode at 0 usually implies the absence or lack of stability of the near-synchrony modes (squares with short delays) and competes with synchrony if it exists. Beyond ϕ_CL_ the recovery intervals become 0, so the stimulus intervals become equal to twice the delay and the region between the vertical dashed lines in **Figure 4B** forms the boundaries for the stable antiphase mode (black circles in the vicinity of c in **Figure 4C**), as the time lags become exactly equal to the delay, and the phase at which an input is received in the stable antiphase model falls on the stable causal limit branch in **Figure 4B**, where the phase is twice the normalized delay. The scaling factor for *k* = 1 antiphase is given by [1 - *f*′(ϕ_AP_)][1 - *f*′(ϕ_AP_)], which is 0 on the causal limit line and implies convergence within a single cycle in the neighborhood of the fixed point.

The antiphase mode loses stability because ϕ_AP_ “wraps around” and falls on the destabilizing left branch of the PRC for delays greater than half the intrinsic period (Woodman and Canavier, 2011). For normalized delays between 0.5 and ϕ_CL_, the leader–follower mode reappears (**Figure 2D** and region near d in **Figure 4C**). For the *k* = 2 leader–follower mode, the sum of the stimulus intervals equal to twice the delay (**Figure 3D**). Using the definition of the stimulus intervals, we obtain that the normalized delay is equal to (ϕ_L_ + ϕ_F_)/2, marked as open circles in the PRC in **Figure 4A**. The horizontal dashed lines show a minimum normalized delay of about 0.5 is required for ϕ_L_ = 0 and ϕ_F_ = 1 + *f*(0), and a maximum normalized delay of ϕ_CL_, beyond which causal limit synchrony emerges as described above. The scaling factor for the *k* = 2 leader–follower mode is 1 - *f*′(ϕ_L_) - *f*′(ϕ_F_). Since *f*′(ϕ_F_) = 1, the scaling factor reduces to -*f*′(ϕ_L_), which is positive. If the latter slope is <1, which it generally is, stability is guaranteed.

**FIGURE 5:**
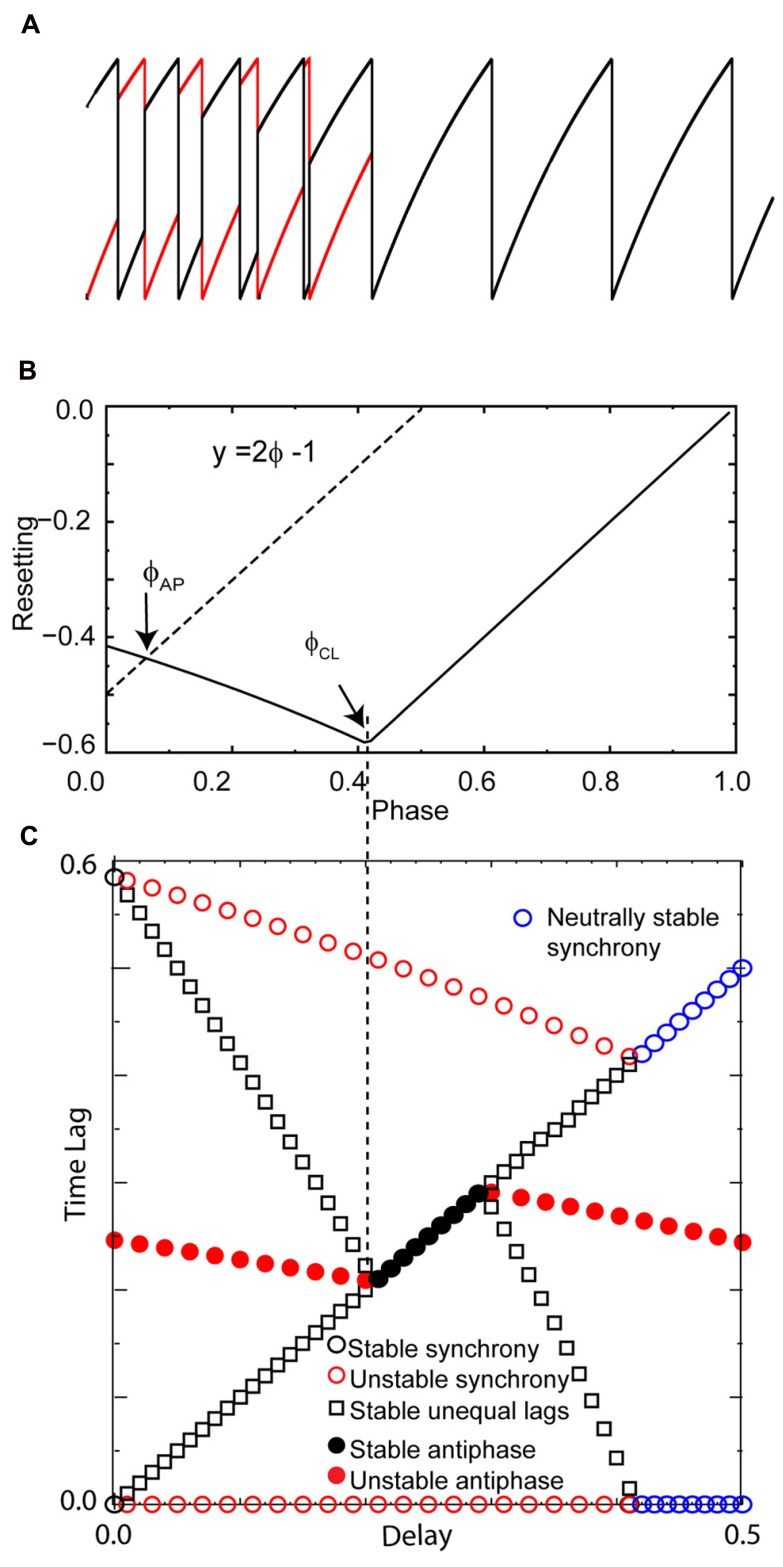
**FIGURE 5.** **Right skew is not rigorously required for zero lag synchrony or leader–follower modes in excitatory pairs.** An extreme example was constructed to show that right skew is not required for these phenomena. Parameters for the coupled LIF pair are γ = 0.9, *S*_0_ = 1, ε = 0.05. **(A)** Convergence to synchrony with no conduction delay. **(B)** Left skewed phase resetting curve enters the causal limit region beyond the phase ϕ_CL_ at about 0.4. Again, the dashed line labeled *y* = 2ϕ - 1 give the input phase for the unstable antiphase mode ϕ_AP_ with zero delay. **(C)** Solution structure for delays less than half the intrinsic period shows a stable leader–follower mode with time lags proportional to the delay emanating from synchrony at zero delay. The symbols have the same meaning as in **Figure 4**, and all quantities are normalized by the intrinsic period.

In order to confirm that our graphical analysis of the PRC yields the correct predictions for modes with unequal time lags (specifically leader–follower modes for the LIF model) regardless of whether the PRC is right or left skewed, as well as to confirm that the stable synchronous modes results from the steep slope at 1^-^ and not directly from right skew, we constructed the counterexample in **Figure 5**. The pulse coupling was made to be very strong in order to extend the causal limit region of the PRC in **Figure 5B** leftward. **Figure 5A** illustrates with one example set of initial conditions that for zero delay, all initial conditions converge to synchrony. Synchrony at zero delay remains stable, and the intersection of the line *y* = 2ϕ - 1 with the PRC that gives the stability of the antiphase mode at zero delay still falls on the unstable branch, and the leader–follower modes with unequal time lag still mediate a gradual transition from synchrony to antiphase as the conduction delays are lengthened. At a delay corresponding to the value ϕ_CL_, the antiphase mode is stabilized. This extreme, artificial example that shows that right skew is not required for synchrony at zero delay nor the gradual transition with near synchronous modes at small delays. However, in the more realistic examples given in the next section, increasing right skew does promote synchrony and near synchrony for excitatory coupling.

### LEFT SKEW STABILIZES ANTIPHASE AT SHORT DELAYS AND PROMOTES BISTABILITY FOR CONDUCTANCE-BASED MODEL WITH EXCITATORY COUPLING, UNLIKE THE LIF RESULTS

**FIGURE 6:**
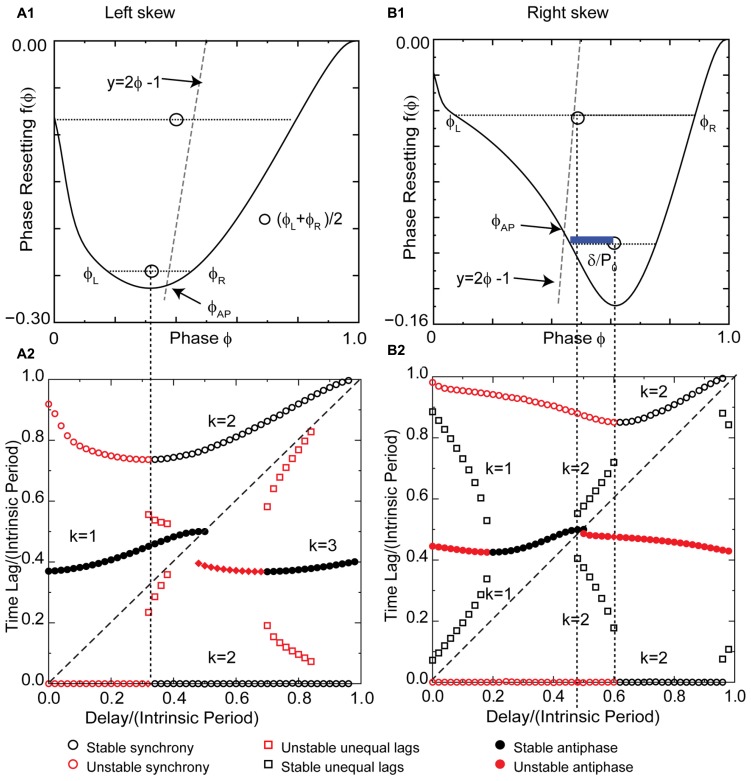
**FIGURE 6.** **Skew influences solution structure of Wang–Buzsaki type 1 excitatory model neuron.** **(A1)** Left skewed type 1 PRC due to excitation. Simulated at *g*_syn_ = 0.06 mS/cm^2^ and *I*_stim_ = 1 μA/ms for both neurons. The intersection of the dashed line *y* = 2ϕ - 1 with the PRC gives the phase for the stable antiphase mode ϕ_AP_ with zero delay. Open circles are the average (ϕ_L_ + ϕ_R_)/2 for pairs of phases on the left and right branches with equal phase resetting. They fall to the left of the dashed line, so there is no leader–follower branch at early phases. **(A2)** Predicted solution structure as delays are varied for two neurons coupled via the PRC in **(A1)**. The two time lags between the firings of the two neurons are represented by a pair of red symbols (unstable mode), or a pair of black symbols (stable mode). Only one symbol is visible for antiphase because the two time lags are equal, indicated by a filled symbol. For synchrony one time lag is 0. **(B1)** Right skewed type 1 Wang–Buzsaki PRC with *g*_K_ = 40 mS/cm^2^. The antiphase mode for zero delay falls on the unstable branch. The open circles that indicate the center between the two branches fall to the right of the dashed line, so there is an unequal time lag branch at short delays (black squares for *k* = 1 in **B2**). The blue bar shows a delay that falls on this branch. **(B2)** Predicted solution structure as delays are varied for two neurons coupled via the PRC in **(B1)**. *k* Values are given for the stable (black) branches.

The pulse-coupled LIF is not very physiological, especially with respect to the instantaneous pulse coupling in the voltage waveform. The generic modes observed in the LIF are modified in networks of real neurons, and their closer analogs, conductance-based models, because a spike in one neuron cannot immediately trigger a spike in another – there must be a finite delay. **Figure 6A1** shows a typical left skewed type 1 PRC for a Wang–Buzsaki model neuron receiving excitatory synaptic input. The PRC has a left branch with a destabilizing slope and a right branch with a stabilizing slope. The vertical dotted line separates the branches. Unlike the extreme example of left skew for a pulse-coupled LIF neuron given in **Figure 5**, the left skew in a more realistic model does not give rise to synchrony with zero delay, nor to the leader–follower branch with near synchrony at small delays. Instead, the synchronous mode is unstable for zero delay because the destabilizing slope at 0^+^ dominates the less steep stabilizing slope at 1^-^. Synchrony remains unstable for normalized delays to the left of the vertical dotted line (red circles to the left of the dotted line in **Figure 6A2**). The leader–follower branch does not emerge at small delays because of the left skew as explained below. One important consequence of the non-zero recovery intervals in realistic models (and real neurons) is that synchrony with normalized delays greater than ϕ_CL_ is stabilized, as opposed to neutrally stable and unobservable as for the case of the pulse-coupled LIF. The slope on the right branch is less steep ensuring convergence because the scaling factor 1 - 2*f*′(ϕ) is guaranteed to have an absolute value <1 for positive slopes <1. Optimal convergence occurs when the slope at the locking point equals 0.5.

**FIGURE 7:**
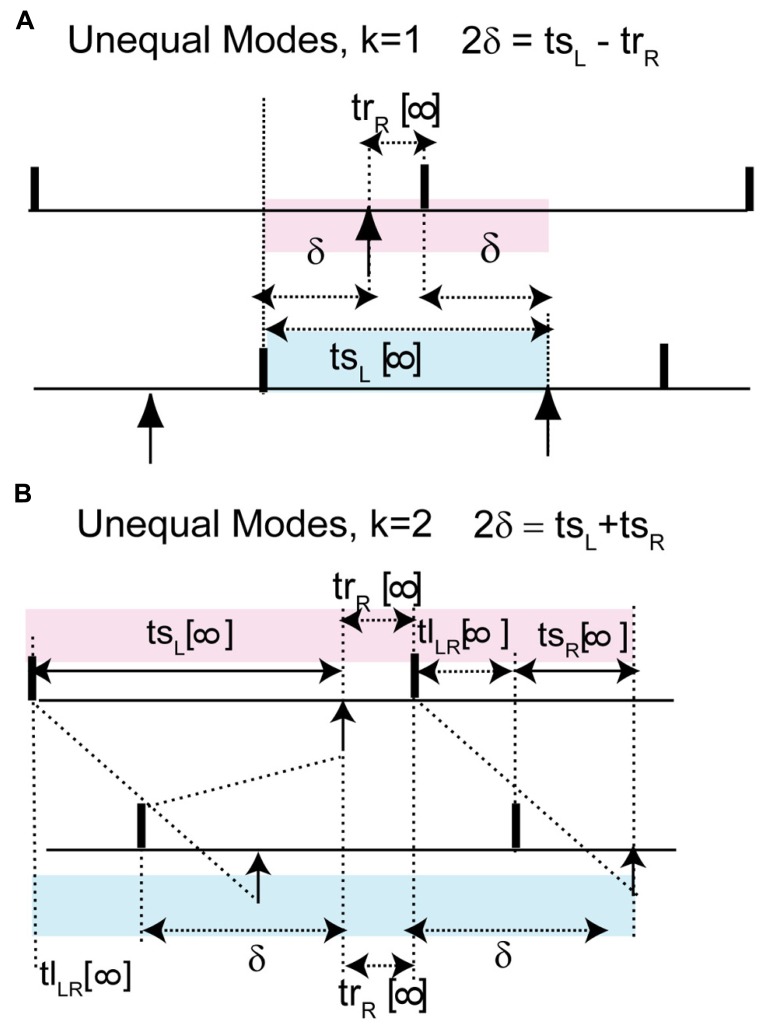
**FIGURE 7.** **The leader–follower modes persists approximately as unequal time lag modes in models with more realistic coupling.** Unlike the leader–follower mode here the recovery interval of the neuron on the right branch is not 0. **(A)** For *k* = 1, 2δ + *t*r_R_ = *t*s_L_, where 2δ + *t*r_R_ is shaded in pink and *t*s_L_ in blue. The normalized delay after substitution of the recovery and stimulus interval can be rewritten as δ/*P* = (ϕ_L_ + ϕ_R_)/2 - [1 + *f*(ϕ_R_)]/2. This gives a criterion for non-negative delay. **(B)** For *k* = 2, 2δ = *t*s_L_ + *t*s_R_. This is obtain by noting that the sum of the pink intervals *t*s_L_ + *t*r_R_ + *t*l_LR_ + *t*s_R_ is equal to the sum of the blue intervals *t*l_LR_ + δ + *t*r_R_ + δ and canceling *t*r_R_ + *t*l_LR_ from both sides. Normalized delay is equal to the average of the phase locking points: δ/*P* = (ϕ_L_ + ϕ_R_)/2.

The most critical result of this paper, which is the effect of skew on the existence of unequal modes, can be explained as follows. The key idea is that the same line that determines the location and hence the stability of the antiphase mode also determines whether a positive value of the conduction delay can support the near synchrony that is part of the leader–follower solution branch. For *k* = 1, for identical oscillators with identical delays, we obtain 2δ = *t*s_L_ - *t*r_R_ (see **Figure 7A**), where *t*r_R_ is recovery interval for the phase locking point on the right branch and *t*s_L_, the stimulus interval for the phase locking point on the left branch of the PRC. Therefore the recovery interval has to be less than the stimulus interval. [Note that if *f*(ϕ_R_) = ϕ_R_ - 1, which occurs when ϕ_R_ falls in the causal limit region and *t*r_R_ = 0, this condition is automatically satisfied, as in **Figures 4C and 5C**.] Substituting for the recovery and stimulus intervals yields a normalized delay δ/*P*_0_ = (ϕ_L_+ϕ_R_)/2 - [1 + *f*(ϕ_R_)]/2, where *f*(ϕ_R_) = *f*(ϕ_L_). Since the normalized delay has to be non-negative, unequal interval modes with *k* = 1 only exist if the average (ϕ_L_ + ϕ_R_)/2, indicated by the open circles in **Figure 6A1**, is greater than or equal to [1 + *f*(ϕ)]/2. Since the phase resetting corresponding to each open circle is given by the *y*-axis value, this condition is satisfied for phases that lie to the right of the dashed line *y* = 2ϕ - 1, the same line that determines the phase ϕ_AP_ for antiphase at zero conduction delay, because along this line ϕ = [1 + *f*(ϕ)]/2. Since all possible circles lie to the left of this line in **Figure 6A2**, no *k* = 1 branch of solutions with unequal time lags emerges. However, the vertical dotted line shows that a *k* = 2 solution branch (red squares to the right of the line in **Figure 6A2** with unequal time lags does emerge at delays equal to (ϕ_L_+ϕ_R_)/2. In fact, it is easy to see that the *k* = 2 unequal times lags (including leader–follower) mode always exists, because it is not possible for the delay to be negative in this scheme. However, this mode is not guaranteed to be stable. **Figure 7B** shows that for unequal time lag modes with *k* = 2, δ - *t*s_L_ = *t*s_R_ - δ which implies that the sum of the stimulus *t*s_L_ and the recovery intervals *t*r_R_ equals twice the delay: 2δ = *t*s_L_ + *t*s_R_. In this case, the normalized delay δ/*P*_0_ is the average (ϕ_L_ + ϕ_R_)/2, which is the same expression as that of the leader–follower mode for *k* = 2. In contrast to the LIF example, this branch of solutions with unequal time lags is unstable because the slope on the destabilizing left branch dominates due to the shallower slope of a right branch that does not fall on the causal limit.

The same line representing *y* = 2ϕ - 1 gives the phase ϕ_AP_ antiphase mode for zero delay at the intersection with the PRC (**Figure 6A1**). The left skew favors the stability of the antiphase mode for zero delay because it extends the stabilizing right branch of the PRC to smaller phases, and this stability persists for a range of delay values (black circles marked *k* = 1 in **Figure 6A2**). Since ϕ_AP_ = [1 + *f*(ϕ_AP_)]/2 + δ/*P*, increasing the delay shifts the antiphase mode rightward. The synchronous solution (*k* = 2) with ϕ = δ/*P* is stabilized by its arrival on the right branch before the antiphase solution reaches the end of the right branch and loses stability as it jumps to the left branch. This overlap enables bistability for some delays. As delays are further increased, the *k* increases to 3 and the generic solutions recur (Woodman and Canavier, 2011). Left skew promotes bistability by increasing the length of the stabilizing branch compared to the destabilizing branch, increasing the likelihood that solutions for different *k* values at the same delay can be concurrently stable.

### RIGHT SKEW FAVORS A GRADUAL TRANSITION FROM NEAR SYNCHRONY TO ANTIPHASE IN CONDUCTANCE-BASED MODELS WITH EXCITATORY COUPLING, SIMILAR TO LIF RESULTS

A right skewed PRC (**Figure 6B1**) was obtained by increasing the potassium conductance. The antiphase mode again emerges for zero delay at the intersection of the line *y* = 2ϕ - 1 (gray line in **Figure 6B1**) with the PRC, but the right skew destabilizes the antiphase mode by causing it to fall on the destabilizing left branch, and the destabilization persists for short delays. The slope on the right branch at 1^-^ is not in the causal limit region, and is insufficiently steep to stabilize synchrony with zero delay. The synchronous solution branch is qualitatively similar to that for left skew. However, the right skew enables the existence of the modes with unequal time lags by the same mechanism that it stabilizes antiphase; shifting the PRC with respect to the line *y* = 2ϕ - 1. In contrast to the open circles representing the average phase (ϕ_L_ + ϕ_R_)/2 of a pair with the same resetting, there are open circles in **Figure 6B1** that lie to the right of this line. The blue bar indicating the phase gap between the line and the open circle gives the magnitude of the normalized delay for that mode. The pairs of black squares in **Figure 6B2** at short delays show that the time lag can be quite short for small delays, so near synchrony can potentially be enabled by right skew in oscillators with type 1 PRCs. These modes are stable because the right skew tends to make the slope on the stabilizing right branch steeper than that on the left, favoring stability by keeping the scaling factor [1 - *f*′(ϕ_L_)][1 - *f*′(ϕ_R_)] below 1. A positive slope (≤1) decreases the magnitude of [1 - *f*′(ϕ_R_)], which compensates for [1 - *f*′(ϕ_L_)] being >1. The unequal modes lose existence at a delay equal to δ/*P*_0_ = (ϕ_L_ + ϕ_R_)/2 - [1 + *f*(ϕ_R_)]/2, where ϕ_L_ = ϕ_R_, exactly the same delay δ/*P*_0_ = ϕ_AP_ - [1 + *f*(ϕ_AP_)]/2 at which the antiphase mode gains stability because ϕ_AP_ also shifts to the stable right branch at the point on the PRC at which ϕ_L_ = ϕ_R_ in a bifurcation that is generic for pairs of oscillators coupled by excitation with right skewed type 1 PRCs. This is evidenced by the pairs of black squares coalescing to a region with only one filled circle visible in **Figure 6B2**.

A *k* = 2 branch of unequal time lag solutions emerges before antiphase loses stability. As in the case of left skew, the average phase (ϕ_L_ + ϕ_R_)/2 of a pair with the same resetting is equal to the normalized delay (see also schematic in **Figure 7B**) as indicated by the vertical dotted line emanating from the open circle in **Figure 6B1** and demarcating the end of the leader–follower *k* = 2 branch in **Figure 6B2**. Unlike the analogous mode for left skew, this mode is stabilized by the steeper slope of the PRC on the right branch compared to the left, again caused by the rightward skew, because the scaling factor 1 - *f*′(ϕ_L_) - *f*′(ϕ_R_) is <1.

### LEFT SKEW FAVORS SYNCHRONY THAT IS ROBUST TO SUBSTANTIAL DELAYS IN PAIRS COUPLED WITH INHIBITION

**FIGURE 8:**
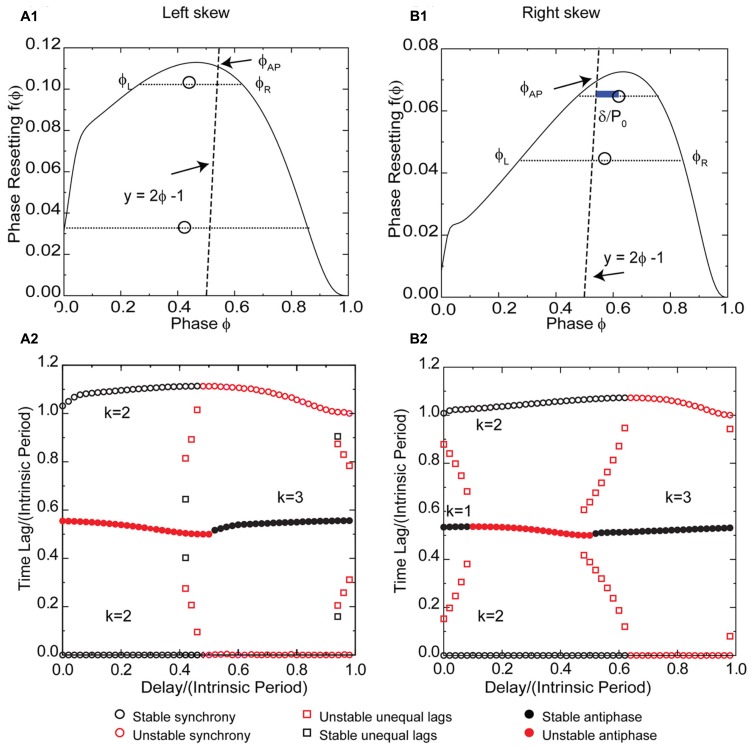
**FIGURE 8.** **Synchrony is robust to conduction delays for skewed type 1 PRCs in response to inhibition, although right skew favors antiphase for small conduction delays.** *k* Values are given for the stable (black) branches. **(A1)** Typical type 1 left skewed Wang–Buzsaki PRC with inhibitory coupling, *g*_syn_ = 0.06 mS/cm^2^ and *I*_stim_ = 1 μA/ms, *g*_K_ = 5 mS/cm^2^. The intersection of the dashed line *y* = 2ϕ - 1 with the PRC gives the phase for the unstable antiphase mode ϕ_AP_ with zero delay. Open circles are the average (ϕ_L_ + ϕ_R_)/2 for pairs of phases on the left and right branches with equal phase resetting, and since they fall to the left of the dashed line, there is no unequal time lag mode at short delays. **(A2)** Predicted solution structure as delays are varied for two neurons coupled via the PRC in **(A1)**. The two time lags between the firings of the two neurons are represented by a pair of red symbols (unstable mode) or a pair of black symbols (stable mode). Only one symbol is visible for antiphase because the two time lags are equal, indicated by a filled symbol. Synchrony is stable for delays less than about half the intrinsic period, and antiphase is stable for delays greater than half the intrinsic period. **(B1)** Type 1 Wang–Buzsaki model right skewed PRC with *g*_syn_ = 0.06 mS/cm^2^ and *I*_stim_ = 1 μA/ms, *g*_K_ = 9 mS/cm^2^. The line *y* = 2ϕ - 1 intersects the PRC on the stable left branch, so antiphase with zero delay is stable. The open circles that indicate the center between the two branches fall to the right of the dashed line, so there is an unequal time lag branch at short delays (red squares for *k* = 1 in **B2**), but it is unstable. The blue bar shows a delay that falls on this branch. **(B2)** Predicted solution structure as delays are varied for two neurons coupled via the PRC in **(B1)**. At the shortest delays, synchrony and antiphase are bistable. The basin of attraction for antiphase is large at zero delay but shrinks with increasing delay until antiphase loses stability.

The effect of potassium conductance on the skew of PRCs measured in response to synaptic inhibition is opposite the effect for excitation. Therefore the potassium conductance was reduced to obtain a left skewed PRC (**Figure 8A1**) for a Wang–Buzsaki model neuron receiving an inhibitory synaptic input. For type 1 PRCs in response to inhibition, the slope of the left branch is stabilizing and the slope of the right branch is destabilizing, which is the opposite of the situation for excitation. Synchrony with zero delay is stable for this example with left skew because the stabilizing slope at 0^+^ is steeper than the destabilizing slope at 1^-^. The robustness of the synchronous solution to delays is striking, as the synchronous solution (pairs of black circles with one time lag equal to 0 in **Figure 8A2**) persists for delay values nearly half the intrinsic period. The scaling factor for early synchrony with *k* = 2 is 1 - 2*f*′(ϕ), where the phase corresponds to the normalized delay, so for small positive PRC slopes, the synchronous mode remains stable in the presence of conduction delays. Intuitively and in contrast to the case for excitation in **Figures 3C and 5C**, the slope at 1^-^ is not required for stability, and the loss of the effect of this slope when conduction delays are introduced does not affect the stability of synchrony. Stability of synchrony is lost only when the normalized delay value exceeds the phase that marks the beginning of the right branch of the PRC with a negative, destabilizing slope.

Since the antiphase mode for zero delay occurs at a phase determined by the intersection of the line 2ϕ - 1 = *f*(ϕ) with the PRC, left skew destabilizes the antiphase mode by extending the unstable right branch to earlier phases such that this intersection occurs on the unstable branch as in **Figure 8A1**. The antiphase mode is unstable for delays up to about half the intrinsic period (indicated by red filled circles in **Figure 8A2** for short delays for *k* = 1) because that is the length of the unstable branch. The same mechanism that destabilizes antiphase prevents the existence of modes with unequal time lags for short delays, and also for the most part destabilizes the *k* = 2 leader–follower branch of unequal time lags at delays near 0.5 in **Figure 8A2**. The open circles in **Figure 8A1** marking the average phase for pairs of phases with equal phase resetting on the left and right branches of the PRC fall to the left of the 2ϕ - 1 = *f*(ϕ), so they correspond to unrealizable negative delay values and the *k* = 1 unequal time lags mode (**Figure 7A**) does not exist. The location of the squares in **Figure 8A2** indicates the delay values for the unequal time lags near a delay of 0.5, but since the destabilizing slope on the right branch is in general less steep than that on the stabilizing left branch, these modes are mostly unstable because the scaling factor 1 - *f*′(ϕ_L_) - *f*′(ϕ_R_) is usually >1. For longer delays, at *k* = 3 the generic solutions recur.

### RIGHT SKEW FAVORS ANTIPHASE AND BISTABILITY FOR SHORT DELAYS IN PAIRS COUPLED WITH INHIBITION

Figure 8B1 shows a right skewed PRC of the Wang–Buzsaki model neuron for the same parameter values as in **Figure 6A1** except for the reversal potential of the synaptic conductance, which is inhibitory for the PRC in **Figure 8B1**. The synchronous branch is qualitatively the same as for the left skewed PRC in **Figure 8A1**, and it is stable for conduction delays up to half the intrinsic period for the same reasons. However, the antiphase solution branch is qualitatively different. Since the antiphase mode for zero delay occurs at a phase determined by the intersection of the line 2ϕ - 1 = *f*(ϕ) with the PRC, right skew stabilizes the antiphase mode by extending the stable left branch to later phases such that this intersection occurs on the stable branch as in **Figure 8B1**. The antiphase mode is stable for delays up to about a 10th of the intrinsic period (indicated by black circles in **Figure 8B2** for time lags of about 0.5 at short delays for *k* = 1) because that is the length of stable branch at phases greater than ϕ_AP_. In contrast to **Figure 6B** for inhibition, here the same mechanism that stabilizes antiphase also enables the existence of modes with unequal time lags for short delays. The open circles in **Figure 8B1** marking the average phase for pairs of phases with equal phase resetting on the left and right branches of the PRC fall to the right of the 2ϕ - 1 = *f*(ϕ), so they correspond to the delay values for the *k* = 1 unequal time lags mode indicated by the red squares in **Figure 8B2** that fall between stable synchrony and stable antiphase at short delays. The blue bar in **Figure 8B1** indicating the phase gap between the line and the open circle gives the magnitude of the delay for that mode. Because the destabilizing slope on the right branch is steeper than that on the stabilizing left branch, the scaling factor [1 - *f*′(ϕ_L_)][1 - *f*′(ϕ_R_)] is < -1, so these modes are unstable. Significantly, the structure of the unequal modes solution with small time lags at early delays transitioning into delays equal to half the network period causes the basin of attraction for synchrony to be quite small for very short delays such that most initial conditions lead to antiphase. However, this effect quickly dissipates with increasing delay and synchrony quickly becomes robust over a significant region of delays as in the case for left skew.

The location of the open circles in **Figure 8B1** indicates the delay values for the unequal time lags for *k* = 2 near a delay of 0.5, but because the destabilizing slope on the right branch is steeper than that on the stabilizing left branch, the scaling factor 1 - *f*′(ϕ_L_) - *f*′(ϕ_R_) exceeds 1, destabilizing these modes as indicated by the second set of red squares in **Figure 8B2**. For type 1 inhibition, right skew rather than left skew promotes bistability, because bistability depends upon lengthening the stable branch and the slopes and synchronization tendencies of the left and right branches of the PRC are inverted compared to excitation. For longer delays, at *k* = 3 stable antiphase recurs. The bottom line is that for type 1 PRCs in response to inhibition, left skew destabilizes and right skew stabilizes the antiphase mode, therefore left but not right skew favors synchrony at short conduction delays.

## DISCUSSION

### SUMMARY

The major result of this paper is to understand how the shape of the PRC determines the generic modes that are observed in pairs of neurons (or other oscillators) with no delays, and how conduction delays affect the tendency of pairs of neurons to synchronize. Specifically, a gradual transition from synchrony to antiphase with increasing conduction delay exists only if the center of the two branches lies to the right of the invariant line whose intersection with the PRC determines the intrinsic phase at which each neuron receives an input in the antiphase mode with no delay. For type 1 PRCs and mutual excitation, right but not left skew enables near synchrony at short delays by shifting the center of the two branches to the right of this invariant line. In contrast, for type 1 PRCs and mutual inhibition, left but not right skew favors synchrony at short delays by destabilizing the competing antiphase mode by causing the intersection with the invariant line to occur on the unstable right branch. We show that exact synchrony with no delay for type 1 inhibitory but not excitatory PRCs is robust to conduction delays, because only the PRC for excitation relies on the stabilizing slope of the PRC at late phases to stabilize synchrony with no delay. A recent experimental study (Wang et al., 2012) confirmed the fragility of the synchronous mode for excitatory synaptic coupling in the presence of conduction delays and the robustness of this mode for inhibition. Generic solution structures are given herein for type 1 PRCs; however, the existence and stability criteria for all generic modes are general and apply to any shape PRC. Consistent with previous work, the effect of skew also manifests itself via differential effects on the slopes of the two PRC branches. Several stability features of the generic solutions for excitatory coupling depend critically on the increase in the steepness of the slope of the PRC at late phases mandated by causality.

### EXTENSION TO OTHER PRC SHAPES

For PRCs with more than two branches, any two branches could in principle give rise to the solutions with unequal time lags that provide a gradual transition between synchrony and antiphase. For example, a type 2 PRC in response to excitation typically has two lobes (Ermentrout, 1996): at early phases the first lobe consists of delays and the second lobe of advances. If the center of the second lobe lies to the right of the invariant line, then modes with unequal time lags and relatively short delays could be enabled and stabilized, so right shift of the extremum of the second lobe would favor such modes. Furthermore, the branch between the maximum advance and the maximum delay is unstable, so shifting the peaks so that the intersection with the invariant line does not fall on this branch removes bistability of antiphase with synchrony at zero delay, favoring synchrony. As the frequency is increased, the first lobe of the type 2 PRC shrinks (Fink et al., 2011). In principle the effect of any PRC shape can be understood by applying the methods described in this study. The only critical assumptions are that each neuron emits one spike for every spike emitted by the partner, that the PRC of each isolated neuron in response to an input from the partner is known, that the PRC still characterizes the response of the neuron to an input received within the coupled network, and that the effect of each input does not persist after the next spike in the same neuron that received the input.

### GENERIC NATURE OF OUR RESULTS COMPARED WITH SPECIFIC MODEL APPROACHES

The three major approaches (Ermentrout and Chow, 2002) to studying coupled oscillators are (1) to study specific model such as the LIF model or the Hindmarsh–Rose model, (2) to use a weak coupling assumption, or (3) to use a pulsatile coupling assumption. We chose to use the latter. Previously, Dhamala et al. (2004) showed that time delays can enhance neural synchrony by calculating the largest Lyapunov exponent for time delayed networks of diffusively coupled Hindmarsh–Rose model neurons. Clearly our methods also illustrate how delays can enhance neural synchrony. For example, in **Figure 6A**, synchrony is unstable for delays less than about a third of the intrinsic period, but is stable for delays from a third of an intrinsic period to an intrinsic period. For a 40-Hz gamma oscillation, regions separated by 8–25 ms would synchronize optimally. Our approach does not rely on knowledge of the differential equations that describe particular neurons, only of the relevant PRC, therefore it is quite general.

Our work on the LIF oscillator was motivated by studies of pulsatile coupling (Ernst et al., 1995, 1998) that extended the results of Peskin (1975) and Mirollo and Strogatz (1990) to the case of two pulse-coupled oscillators reciprocally coupled with delays up to half the intrinsic period. Their assumptions implicitly defined a PRC and allowed the construction of return maps. The stable fixed points of these maps revealed that for small delays and strong excitatory coupling, at all coupling strengths synchronization with a phase lag equal to the delay was found to be always stable, analogous to our leader–follower mode. For inhibition, bistability between synchrony and antiphase was observed but as the coupling strength was increased only synchrony remained. Zeitler et al. (2009) studied the bifurcation structure of pairs of similar oscillators also coupled via conduction delays and noted that for these systems, only antiphase and synchrony could be stable for identical, identically coupled oscillators, but modes with unequal time lags could acquire stability in pairs coupled by excitation, similar to what we have found. The solution structure of simplified models such as the pulse-coupled LIF is not always representative of that obtained for conductance-based models. Real neurons (and conductance-based models) can exhibit much more nuanced PRCs, and the theoretical framework presented here includes and expands previous work on pulse-coupled oscillators with delay.

### DIVERGENT PREDICTIONS OF PULSATILE COUPLING THEORY VERSUS WEAK COUPLING THEORY

Weak coupling theory cannot be used to analyze pulsatile coupling of the type proposed by Peskin (1975) and Mirollo and Strogatz (1990), in which a finite and constant perturbation in voltage results from a presynaptic threshold event, with the caveat that an increase in the postsynaptic membrane potential beyond threshold has no additional effect. The PRC for an infinitesimal perturbation in membrane potential (equivalent to an infinitesimal perturbation in membrane current) has been derived for the LIF oscillator (Brown et al., 2004), and the PRC given in Eq. 1 for strong coupling cannot be derived from the infinitesimal PRC. Furthermore, the stability results for weakly coupled LIF oscillators and pulse-coupled oscillators are not in agreement. Weakly coupled type 1 oscillators do not synchronize with excitation (Hansel et al., 1995), but pulse-coupled oscillators synchronize both for the two oscillator circuit (Peskin, 1975) and all-to-all coupled circuits of *N* oscillators (Mirollo and Strogatz, 1990). Weak coupling does not account for the increase in the slope on the right branch imposed by causality as the conductance is increased, but instead assumes the PRC scales with increasing coupling the same way at all phases. This is an important limitation of weak coupling theory that has not been previously documented, and applies to synchrony in all circuits of oscillators that have PRCs with a destabilizing slope at a phase of 0 but a stabilizing slope at a phase of 1. Another disagreement between weak coupling theory and strongly pulse-coupled theory is that weak coupling (for example, Ladenbauer et al., 2012) assumes that in the presence of delays, synchrony always exists with both neurons receiving an input at zero phase, but clearly for oscillators coupled by strong excitation, at sufficiently long delays, the synchronizing input actually occurs at a late phase on or near the causal limit region of the PRC. Finally, weak coupling does not recognize how the stability criterion changes with the duration of the feedback loop.

### FUNCTIONAL SIGNIFICANCE: PRC SKEW AND THUS SYNCHRONIZATION PROPERTIES CAN BE MODULATED

Ermentrout et al. (2012) proposed that modulation of intrinsic ion channels could quickly reverse the synchronization tendencies of neurons by altering the PRC shape, providing a switch to turn synchrony on and off rapidly. There are several ways in which altering the conductances (Ermentrout et al., 2001, 2012; Pfeuty et al., 2003; Gutkin et al., 2005; Stiefel et al., 2009) can change the shape of a type 1 PRC for a regularly spiking neuron. Reducing restorative potassium currents or increasing regenerative sodium currents favors left skew if these currents are active at rest, whereas manipulations in the opposite direction favor right skew. This principle was used to manipulate the skew of the PRC in the Wang–Buzsaki model neuron used in this study. For the baseline potassium conductance value *g*_K_ = 9 mS/cm^2^, the PRC for the Wang–Buzsaki model neuron in response to excitation was left skewed but the PRC in response to inhibition was right skewed. For excitation, *g*_K_ was increased to change the PRC skew from left to right, and for inhibition, the *g*_K_ was decreased to change the skew from right to left. Taken to the extreme, manipulations of currents active at rest that favor right skew can change the underlying bifurcation and PRC type from 1 to 2 (Ermentrout et al., 2001; Prescott et al., 2008; Stiefel et al., 2008), which often changes the stability by changing the sign of the slope at zero phase. On the other hand, manipulations of currents that are only activated by spikes cannot in general change the PRC type, but they can alter its shape (Ermentrout et al., 2001, 2012; Gutkin et al., 2005). Ermentrout et al. (2001) also showed that adding either recurrent inhibition or adaptation with a sharp, depolarized threshold such that it was only evoked by spikes, preserved the type 1 character of the PRC but shifted the skew to the right as expected for increases in outward current. However, an exception to this general pattern was found in which increasing an outward current that contributes to the afterhyperpolarization following a spike promoted left rather than right skew, because the primary effect of the change was to increase sodium channel availability (Ermentrout et al., 2012). Thus, there are many plausible modulatory targets available for changing the synchronization tendencies of biological networks.

### PREVIOUS STUDIES EXAMINING SKEW IN THE CONTEXT OF WEAK COUPLING WITH NO DELAY

Weak coupling (Ermentrout and Kopell, 1990, 1991; Ermentrout, 2002) identifies one to one phase-locked modes in identical coupled pairs by finding the zero crossings of *H*(ϕ) - *H*(1 - ϕ), in which the *H* function is equivalent to our phase resetting *f*(ϕ) except opposite in sign. Instead of neglecting changes in frequency caused by the coupling, our method finds the equivalent of the zero crossings of *H*(ϕ) - *H*[1 - ϕ - *H*(ϕ)], which contains an extra *H* function within the argument of another *H* function in order to update the elapsed time by the non-negligible resetting in the partner neuron. One consequence of neglecting the contribution of phase resetting to the network period is that for weak coupling, antiphase is always assumed to occur at an intrinsic phase of 0.5 instead of 2ϕ_AP_ - 1 = *f*(ϕ_AP_). A critical role for PRC skew in networks of type 1 neurons connected by mutual synaptic excitation was demonstrated by Ermentrout et al. (2001), who showed that for certain model neuron pairs with slightly right skewed type 1 PRCs in response to excitation, both synchrony and antiphase were unstable, and near antiphase was the only stable solution. They used weak coupling to explain their results, and plotted *H*(1 - ϕ) - *H*(ϕ) to get the phase-locked modes from the zero crossings and the stability from the slope *H*^′^(1 - ϕ) - *H*^′^(ϕ), which must be >0 for stability. The stability criterion is slightly different than the ones we utilize because changes in the network period due to resetting are neglected as explained above. Nonetheless, the stability analysis is usually quite similar, for example, the stability of synchrony is determined entirely by whether the slope of the PRC is steeper before or after the spike, with the former case implying stability for the case of pairs of neuron coupled via type 1 PRCs in response to excitation. Ermentrout et al. (2001) then skewed type 1 PRCs farther to the right by flattening the slope at early phases while increasing the steepness at late phases. The increased skew caused the stable zero crossings to shift toward near synchrony, in which one neuron of the pair fires just before the other, as shown in our **Figure 6B**. Our extension of their work is that we explain directly in terms of the shape of the PRC how the stabilization occurs by bringing the unequal time lags solution branch into existence.

Ermentrout et al. (2012) also give an example of a different stabilization mechanism for a pair of Golomb and Amitai (1997) model neurons with reciprocal synaptic excitation and type 1 PRCs in which exact synchrony is stable for the baseline parameters given because of the right skew of the PRC. In this mechanism the right skew preferentially steepens the stabilizing slope at 1^-^ compared to the nearly flat destabilizing PRC slope at 0^+^. Pfeuty et al. (2003) had complementary results showing that skewing the PRC toward the left stabilized the antiphase mode for two mutually electrically coupled neurons by causing the antiphase mode near a phase of 0.5 to fall in the region of stable slope. Similarly, Zahid and Skinner (2009) showed that for pairs of electrically coupled neurons, right skew favors small phase lags because both synchrony and antiphase were unstable for the type 1 PRCs they observed, but sufficient left skew can stabilize antiphase and cause it to be globally attracting. In that study, skew was quantified by the fraction of the area under the PRC that fell to the left of a phase of 0.5, and weak coupling theory was invoked to show how destabilization of the antiphase mode by right skew led to the emergence of nearly synchronous modes with one small time lag. Electrical coupling is more analogous to excitation than inhibition in spiking neurons if the effect of the depolarizing effect of the suprathreshold spike dominates (Chow and Kopell, 2000), so these results are consistent with the framework presented in this paper. The advance in theory presented in this paper is that we do not make the weak coupling assumption, but instead show graphically that the destabilization of antiphase mode and the emergence of near synchrony depends on the location of the peak of the PRC relative to the location of the line that gives the phase of the antiphase mode at zero delay in terms of the intrinsic period.

### PREVIOUS STUDIES EXAMINING SKEW IN THE CONTEXT OF WEAK COUPLING WITH CONDUCTION DELAY

Remme et al. (2009) examined oscillatory dendritic compartments separated by passive cylindrical dendritic compartment of different electrotonic lengths, somewhat analogous to introducing a delay. Under weak coupling assumptions, they found that a left skewed PRC, or interaction function *H*(ϕ), yields bistability between synchrony and antiphase, whereas a right skewed interaction function yields gradual transitions between the two modes as the delay was increased. Again, results for electrical coupling parallel our results for synaptic excitation in **Figure 6**. Ladenbauer et al. (2012) also showed that increasing right skew in pairs of type 1 neurons coupled by synaptic excitation favored smaller phase lags decreasing to 0 at no delay, and favored the leader–follower mode by destabilizing the antiphase mode in the presence of conduction delays. For inhibition, increasing right skew stabilized the antiphase mode and promoted bistability with synchrony that persisted with short conduction delays. The weak coupling analysis of an adaptive exponential integrate and fire neuron (aEIF) in **Figure 6** of that paper is consistent with our **Figures 6 and 8**.

### EFFECT OF DISCONTINUITIES

The criteria for exact synchrony given in this paper are only strictly valid if there is no resetting in the cycle following the perturbation (Oprisan et al., 2004; Achuthan and Canavier, 2009), called second order resetting. Second order resetting is most prominent for inputs given just before a spike, so adding conduction delays for the most part precludes receipt of an input just before a spike and minimizes the importance of second order resetting. A complete treatment of stability with discontinuities must take into account that the first order phase resetting at a phase of 1 is 0 because an input applied after the cycle is over cannot affect that cycle. Effects of discontinuities are treated in Ladenbauer et al. (2012), Dodla and Wilson (2013), and Wang et al., (2012, supplementary material).

### IMPLICATIONS OF GENERIC MODES FOR LARGER NETWORKS

Some of the results presented herein may also be extendable to networks of all to all connected neurons. For type 1 PRCs in response to excitation, the right branch of the PRC tends to stabilize synchrony, since if a neuron spikes later than the group, it receives an input at a late phase (1^-^, just to the left of 1) that advances it more than the group on the next cycle bringing it closer to synchrony. On the other hand, the left branch tends to destabilize, since a neuron that spikes before the group receives an input at an early phase (0^+^, or just to the right of 0) that advances it more than the group, taking it farther from synchrony. Simulations of pulse-coupled LIF neurons (Ernst et al., 1995; Coombes and Lord, 1997) have previously shown that the globally attracting synchronization of *N* pulse-coupled oscillators (Peskin, 1975; Mirollo and Strogatz, 1990) with type 1 PRCs comprised of all advances (excitation) is easily disrupted by conduction delays. Therefore the population activity is predicted by the activity of a single pair, in which synchrony is also disrupted by conduction delays.

Another possible extension is to clustering in larger networks. Bistability between synchrony and antiphase supports clustering (Gutkin et al., 2005; Jeong and Gutkin, 2007; Achuthan and Canavier, 2009). Chandrasekaran et al. (2011) have shown how two clusters that fire even slightly out of phase with each other can enforce synchrony within each cluster, even if exact synchrony within the isolated cluster in unstable, so this mechanism should generalize to enforce near synchrony in larger networks with one cluster firing slightly before the other. The leader–follower mode has been shown to stabilize clusters to some degree in networks with delay (Ernst et al., 1995). The strongly stabilizing effect of the causal limit region of excitatory PRCs is only adequately considered using the methods for strong coupling described herein.

The most important extension of these results is to synchronization between distal brain regions. Previously it was thought that long projections connecting brain regions were excitatory, but recently long distance inhibitory connections have also been identified (Melzer et al., 2012). For two mutually coupled populations in two different brain regions, the results from this study and our previous study (Wang et al., 2012) show that inhibitory projections may more reliably synchronize these populations in the presence of conduction delays between distal regions, and that some heterogeneity and noise can be tolerated. Alternatively, if the connections are excitatory and the PRCs type 1, then right skew in the PRC is likely required during episodes of near synchrony. If the unit oscillator is not a single neuron, but rather a network oscillator, the relevant PRCs for the network oscillation can be measured and analyzed for synchronization tendencies in a similar fashion to that for a single neuron (Akam et al., 2012).

A final possible extension relates to the dynamic relay hypothesis which suggests that synchronization among distal neurons can be achieved via symmetric coupling through a hub neuron. Viriyopase et al. (2012) studied the simplest such system with two outer neural oscillators each reciprocally connected to a third neuron, the relay neuron via identical reciprocal delays. They identified a “pacemaker” regime in which all three neurons fired simultaneously in the causal limit synchrony mode, that is, all neurons fired immediately upon receiving delayed input from the neuron or neurons to which it is connected. They also identified two other modes, “slave synchrony” in which the outer neurons were leaders and the relay neuron was a follower, and a “driven synchrony” mode in which the converse was true. Therefore the concepts developed herein for two neurons are directly extendable to *N* neurons each reciprocally connected to a hub (but not directly to each other).

## Conflict of Interest Statement

The author declares that the research was conducted in the absence of any commercial or financial relationships that could be construed as a potential conflict of interest.
